# New task–new results? How the gaze cone is influenced by the method of measurement

**DOI:** 10.3758/s13414-024-02884-9

**Published:** 2024-05-09

**Authors:** Linda Linke, Gernot Horstmann

**Affiliations:** Department of Psychology, Bielefeld University, Bielefeld, Germany

**Keywords:** Measurement method, Gaze cone, Overestimation bias

## Abstract

Perceiving direct gaze — the perception of being looked at — is important in everyday life. The gaze cone is a concept to define the area in which observers perceive gaze as direct. The most frequently used methods to measure direct gaze threshold fall into two broad groups: First, a variant of the method of constant stimuli, firstly introduced by Gibson and Pick (*The American Journal of Psychology, 76*, 386–394, 1963). Second, a variant of the method of adjustment, firstly introduced by Gamer and Hecht (*Journal of Experimental Psychology: Human Perception and Performance, 33*, 705–715, 2007). Previous studies found a considerable range of thresholds, and although some influences on thresholds are already known (uncertainty, clinical groups), thresholds often vary for no apparent reason. Another important method is a triadic gaze-perception task, which usually finds triadic gaze direction judgments to be overestimated. In two experiments, we compare the method of adjustment with the method of constant stimuli. Experiment 1 additionally examines the influence of the overestimation effect found in the triadic task. Results indicate that thresholds are larger when measured by the method of adjustment than by constant stimuli. Furthermore, Experiment 1 finds a nonlinear overestimation factor, indicating that gaze directions near 0° are less overestimated than larger eccentricities. Correcting the thresholds with individually obtained overestimation factors widens the gaze cone but does not eliminate the average difference between the methods of adjustments and constant stimuli.

## Introduction

The area of direct gaze—also known as the gaze cone (Gamer & Hecht, 2007)—can be measured by a range of different techniques. The first study on this topic was presented by Gibson and Pick (1963). They measured direct gaze by having a real person looking at certain points on and near the face of the participant with a simple task: “If you are looked at, say yes—if not, say no.” Since that first experiment, the number of studies has increased and is still increasing, and other methods of measurement have been added. With more studies and more methods, the picture had become increasingly rich, but did it also get better, or clearer?

In the field of gaze cone research, the results of different studies have shown some variability with respect to gaze cone width. Conceptually, the perceived gaze cone certainly is expected to show some variability in a population, as any trait or state does. Indeed, some variables have already been identified that may temporarily alter the width of the gaze cone, such as social anxiety or social exclusion (Gamer et al., 2011; Lyyra et al., 2017). On the other hand, measurement techniques may also have had effects, and indeed, a number of methods have been used in the past.

The most frequently used methods in gaze perception research can be divided into two broad groups of tasks: The first task was introduced by Gibson and Pick (1963) and used the method of constant stimuli. These authors presented seven different gaze directions that had to be evaluated by an observer as direct or averted. The task involved only the looker and the participant. As the participant is at the same time the object and the observer of the looker’s gaze, this design has aptly been termed a dyadic variant of a gaze perception task, in contrast to a triadic task, where the looker’s gaze is directed on a third object that is independent from the observer. The distribution of yes answers over the seven gaze directions was obtained and used to extract a measure of threshold for direct gaze. Gibson and Pick (1963) found a threshold of somewhat less than 3° at a distance of 2 m, implying a cone width of about 6°. Please note that Gibson and Pick originally thought that their results could be traced back to an accuracy threshold rather than to an actual area of direct gaze. The latter notion was introduced years later by Gamer and Hecht (2007). Lobmaier and colleagues (2021) found a 5° wide cone at 60 cm distance with the same method, a width which is well above the human accuracy threshold. Balsdon and Clifford (2018) used an elaborated variant of the task and asked participants whether the gaze is orientated left, right, or directly at them, and found a 9° wide cone at a distance of 57 cm.

The second method was introduced by Gamer and Hecht (2007; see also Horstmann & Linke, 2021), who used a variant of the method of adjustments, where the observers adjusted the gaze of the looker to the point where they felt just (not) looked at. This method has proven versatile also in the assessment of gaze cone size in special subpopulations (such as social anxiety, as in Gamer et al., 2011). Gamer and Hecht (2007, Experiments 1–3) found a gaze cone width of 9° at a distance of 1 m, while a distance of 5 m has led to a cone of only 8°, but with no significant difference between the two distances (in their Experiment 4, the gaze cone measure was notably smaller). Gamer and colleagues (2011) found a 11° wide cone for healthy control subjects at 1 m viewing distance, and Harbort and colleagues (2013) found an even wider cone of 14° in their healthy control group, at 1 m viewing distance. While Gamer and Hecht as well as the other studies just mentioned exclusively used their decentering task to define the width of the gaze cone, Horstmann and Linke (2021) used ascending and descending series (i.e., starting in the center and adjusting to the periphery, or starting in the periphery and adjusting to the center) to better control for possible anchor or hysteresis effects. They found a gaze cone of 5°. They also tested a wider range of distances, which did not differ significantly from each other, similar to the majority of studies that varied distances (for an overview of previous studies and their results, see Appendix Table 1).

Based on the two measures, one may observe a slight tendency towards a larger cone width with the method of adjustment than with the method of constant stimuli. One might suspect that the question “are you being looked at” does not evoke the same understanding in observers as the task “adjust the eyes until you are being looked at” or “adjust the eyes until you are no longer being looked at.” Different instructions can lead to different mental representations of aspects of the task for the observers (e.g., Müller et al., 2018).

Another question that remained to be clarified is the influence of the overestimation effect on the perception of direct gaze. The overestimation effect was first described by Anstis and colleagues (1969). These authors used a triadic measurement task that involves three constituents: the looker, the observer, and the object that is looked at by the looker. More precisely, observer and looker sit face-to-face with a bar between them. The looker looks at prespecified points at his side of the bar, and the observer, who has a scale on his side of the bar, has to indicate the position where he perceives the looker’s fixation point. The data can be analyzed by regressing subjective position depending on the objective position, and the slope of the linear equation gives the overestimation factor: Slopes above 1 indicate an overestimation, while slopes below 1 indicate an underestimation; a slope of 1 would be perfect perception. In Anstis et al.’s study, observers overestimate the presented gaze direction by an average of 50–86 %.

Is there a relation between dyadic and triadic tasks? Assuming that performance in both tasks share common perceptual mechanisms, one might suspect that the overestimation effect plays a role in both tasks. If so, the overestimation effect impacts on the gaze cone size. Given that, firstly, the gaze direction is calculated and, secondly, a judgment about the directness of gaze is made, the overestimation may influence the perception of direct gaze. Therefore, an overestimation or underestimation effect could make a gaze direction appear more direct or more averted and could potentially lead to a narrower or wider gaze cone.

There are, however, some differences between the two tasks that could make it difficult to compare a dyadic and triadic method: A dyadic task necessarily involves only two components: The looker and the observer. Here, the observer acts as the object of gaze and as the one who judges gaze. Therefore, the task combines the self-perception of the observers as well as a following judgment if a gaze is directed at them. In contrast, a triadic task involves three components: The looker, the observer, and a third object that is looked at by the looker. Although both tasks involve the same stimulus (the lookers’ eyes), there are some differences in the available information: First, in a triadic task, the size of the third object, which is looked at by the looker, is directly available for the observer. Therefore, the decision whether the gaze direction is in line with the third object can be inferred from the direct visual cue from the object’s position and size. Contrary, in a dyadic task, the size of one’s own body or own face must be inferred indirectly from memory or the body representation. Second, the triadic task includes three important distances: looker-object, object-observer, and looker-observer. Whereas looker-object and observer-object distance can (but does not have to be) be equal, the looker-observer distance has to be different from the previous two mentioned distances. In contrast, the dyadic task involves only one distance: the looker-observer distance. Although it seems reasonable that triadic and dyadic tasks share some common perceptual mechanisms, it is still unclear how distance—as well as self-perception—influence the two tasks.

Balsdon and Clifford (2018) had made a first approach to measure the influence of the overestimation effect on the gaze cone by combining a direct gaze-judgment task (e.g., Gibson & Pick, 1963) and a gaze reproduction task (e.g., Anstis et al., 1969), where participants manipulated a virtual eyeball to match the perceived gaze direction. In some trials, they asked participants to decide whether the presented gaze direction is left, right, or direct (modified gaze-judgment task). In other trials, they asked participants to adjust a “pointer,” which was a virtual sphere that could be rotated to adjust the horizontal angle of the central target, in the direction of the presented gaze direction (modified gaze-reproduction task). It allows to recalculate gaze cone width while accounting for the error that is evident from the matching task. The results of Balsdon and Clifford’s study showed a 1.5 times larger gaze cone size when correcting for the error from the matching task than for uncorrected values.

To summarize, the influence of the different tasks on gaze measurements as well as the influence of the overestimation effect on the perception of direct gaze have not yet been empirically addressed. On the one hand, dyadic tasks should ideally reveal the same thresholds when everything else is unchanged. Therefore, the current study aims to answer the question: Do different measurement methods result in consistent measurements of gaze cone width within subjects? In the first experiment, we used a real looker and tested if there is a difference in the average gaze cone width, and how the gaze cone width is influenced by the overestimation effect. The second experiment tested a computer avatar with the methods of adjustment and constant stimuli, and additionally investigated whether there is a correlation between the gaze cone widths measured with different methods under controlled circumstances using a computer avatar.

## Experiment 1

Experiment 1 involved three methods from previous research on gaze perception: the methods of constant stimuli and of adjustment, and the triadic task. Each observer performed all three measurement methods—one per task. The order of the tasks was counterbalanced across subjects to control for possible serial order effects. Between the different tasks, the apparatus was changed according to the following task.

### Participants

Twenty observers (10 male, 10 female), aged between 16 and 36 years, volunteered for course credit or candy. We assumed a large effect (*d* = 0.7 or higher) even for small mean differences between paradigms. A power analysis using this effect size yielded a target-sample size of *n* = 18 for our critical hypothesis *t* test with the standard 0.8 power, and alpha of 0.05. All observers had normal or corrected-to-normal visual acuity and intact color vision. Participants gave written informed consent before participation. The experiment was approved by Bielefeld University’s ethics committee.

### Task 1: Method of constant stimuli

### Method

#### Apparatus and stimuli

**Fig. 1:**
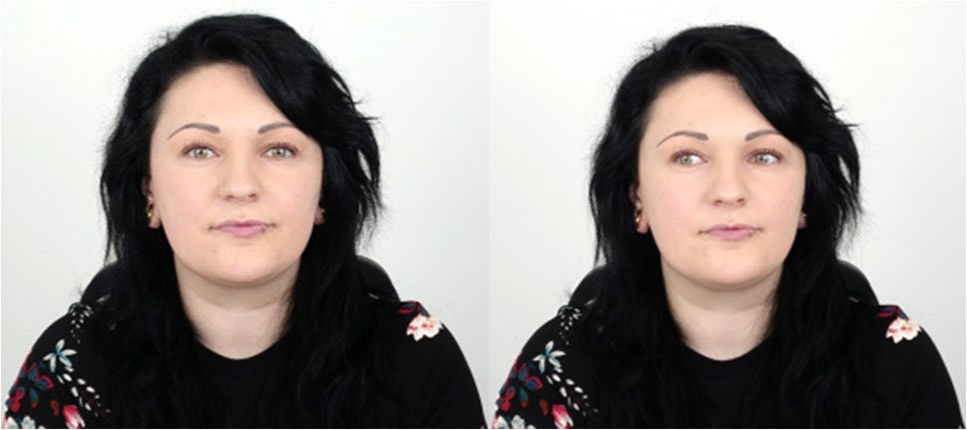
Exemplary photos of the looker model. The left picture shows the looker gazing 0° straight. The right picture shows the looker gazing 13° to the left (from observer’s perspective)

**Fig. 2:**
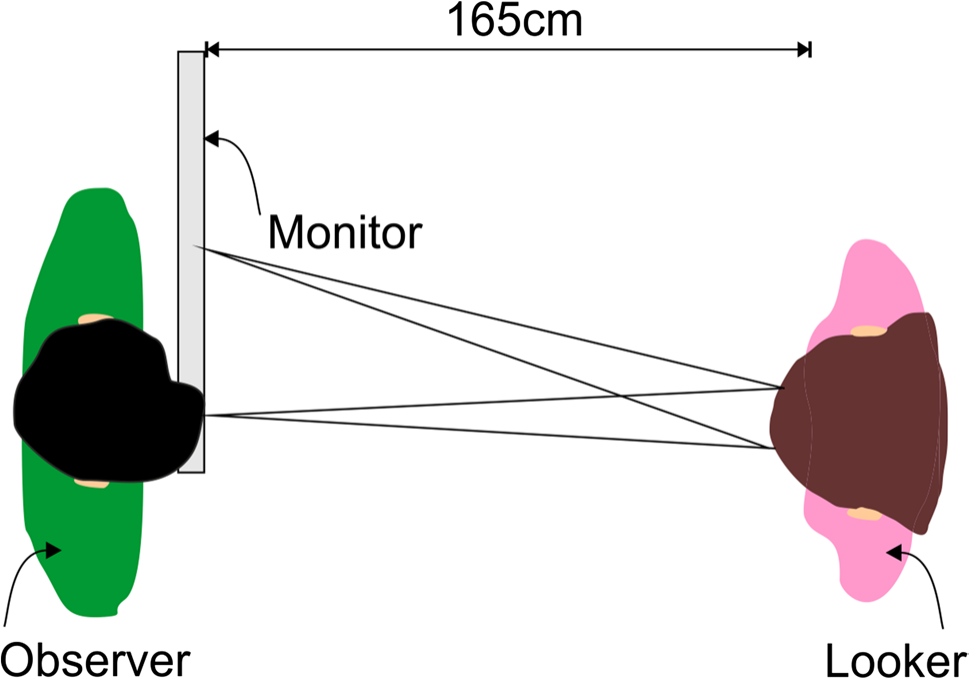
An illustration of the experimental setup. The looker fixated on multiple points on the monitor, which was placed right in front of the observer

A real looker served as the stimulus. The real looker was a 23-year-old woman with normal visual acuity and green eyes (see Fig. 1). Her interpupillary distance was 6.5 cm. She sat at a distance of 165 cm from the observer. The observers sat very close to the backside of a computer monitor, with the upper edge immediately below their eyes. The front side of the monitor was faced toward the looker. Only the upper 5% of the monitor screen was used to present the fixation points for the looker, which thus appeared directly under the observers’ eyes. Because of the narrow frame of the screen, the fixation points appeared actually 2 cm below the eyes of the observer. At a distance of 165 cm, this 2 cm deviation corresponds to a downward shift of the gaze of 0.69°. Studies on acuity of gaze perception suggest that this gaze deviation is not or just barely perceivable by the observer (Gibson & Pick, 1963; Symons et al., 2004). Even if observers can perceive the deviation, it is likely that they nevertheless would perceive the gaze is being directed at them due to the properties of the vertical dimension of the gaze cone (Horstmann & Linke, 2022). Fixation points were white circles with a size of 20 pixels on a light grey background. They were presented on a 51 cm × 35 cm-sized Fujitsu Siemens monitor with a frame rate of 89 Hz. The display had a resolution of 1,680 × 1,050 pixels. The stimuli were presented in full-screen mode. The presentation of the fixation points and the response registration of the observers were controlled by a custom written Python script using routines from PsychoPy (Peirce, 2008). Observers indicated their answers by using the keyboard’s left (for yes) and down arrow (for no) buttons. Gaze directions of the real looker varied between 0° and 13.9° which refers to distances of 0 cm to 39 cm on screen in steps of an exponential function with 2.5 cm. This led to five gaze directions (0°, 0.9°, 2.2°, 5.4°, 13.9°) in total. For reasons of economy only one side of the gaze cone (the left side, from the vantage point of the observer) was measured (see Fig. 2). The symmetry of the gaze cone allows this option (see Gamer & Hecht, 2007; Horstmann & Linke, 2021).

#### Design and procedure

Each trial followed the same sequence of events. First, the observers were asked to close their eyes. The looker then fixated on a fixation cross in the middle of the screen until the fixation point on the top of the screen was presented. Then, the looker fixated on the fixation point, which was either 0°, 0.9°, 2.2°, 5.4°, or 13.9°. Subsequently, the observers were allowed to reopen their eyes. Their task was to judge whether they are looked at or not. They indicated that they are looked at by pressing “yes” (left arrow button) or “no” (below arrow button) on the keyboard. After the observer had pressed the answer key, the next trial began with the presentation of the central fixation cross. The fixation cross and the fixation points were visible only for the looker, but not for the observer. The observer thus only saw the looker’s gaze direction, but not the target of the gaze. Every gaze direction was presented 25 times in random order, which resulted in a total number of 125 trials. The observer performed two practice trials at the beginning.

### Results and discussion

**Fig. 3:**
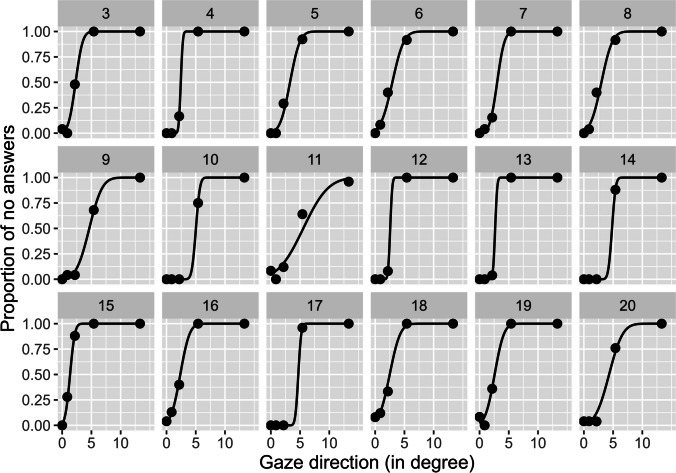
The individual proportions (points) for judgements of direct gaze and the fitted cumulative Gaussians (lines). The *x*-axis depicts gaze direction; the *y*-axes the proportion of averted gaze judgments

The data of 18 observers could be analyzed. Due to an adjustment of the experimental setting after the second observer, the first and second observer had to be excluded. The data were aggregated by observer and gaze direction. Yes and no judgements were inverted to allow the fitting on a cumulative Gaussian function. By using the “quickpsy” package (Lineares & López-Moliner, 2016), an individual fitting per observer was computed. Fitting parameters for all observers were extracted. The 50% point on the fitted curve was used as the threshold of direct gaze. The threshold of direct gaze was 3.33°. Please note that the threshold of direct gaze is only half cone width; two-sided cone width would therefore be 6.6°. Observer’s thresholds varied between 1.35° and 5.54° (see Fig. 3). The data were similar to previous studies using the method of constant stimuli.

### Task 2: Method of adjustment

### Method

#### Apparatus, stimuli, and design

The apparatus was similar to Task 1. The real looker (the same as in Task 1) fixated on a white fixation point on a monitor. In contrast to Task 1, however, the fixation points did not appear as constant stimuli but were continuously adjustable by the observer. The fixation points could be adjusted in two ways: In descending series, the start dot started on the left side of the screen at 0° (0 cm) of gaze direction. As this point was exactly at the center of the observer’s face, it is referred to as the center position. This fixation point could then be adjusted continuously (actually in steps of 0.05°) by using the scroll function of the mouse in smooth movements to the right side of the screen to the edge position (13.9°, 39 cm screen distance). Alternatively, in ascending series, the fixation point first appeared at the edge position—the right side of the screen (39 cm–13.9°), and could be adjusted in smooth movements to the left side in direction to the center position (0 cm, 0°). The looker followed the fixation point with his eyes, appearing as a smooth eye movement to the observer.

Presentation and response registration were controlled by a custom written Python script using routines from PsychoPy (Peirce, 2008). The instruction for a given trial was presented verbally before each trial by the experimenter. Observers were asked to adjust the looker’s eyes until they just felt looked at in ascending series or until they just felt not looked at in descending series. The observers were free to adjust the eyes as long as they wanted to and could correct their adjustments to the left as well as to the right. They confirmed their final result by pressing the “enter” key on the keyboard. By scrolling upward, the fixation point was moved so that the gaze shifted to the right, while scrolling downward led to a gaze shift to the left. Between the trials the observers were again asked to close their eyes and were allowed to open them again when the looker had fixated on the first position of the fixation point of the next trial.

Two warm-up trials were made before the experiment proper, during which the researcher probed the participants’ understanding of the task and provided answers to possible questions. Descending and ascending series were presented randomly and were repeated six times, which resulted in 12 trials per gaze position.

### Results and discussion

The data were averaged for each observer and each of the two series (ascending and descending) separately. Ascending series yielded a mean of 5.45°, with a range between 1.65° and 9.84°, while descending series yielded a mean of 5.08°, with a range between 2.41° and 9.33°. Averaging over both series resulted in a threshold of direct gaze of 5.24°, and a between subject standard deviation of 1.28°. Within-subject standard deviation ranged from 0.38° to 2.66° with an average of 1.05°. A *t* test indicated no significant differences between the two series, *t*(17) = 0.53, *p* = 0.60, and a small non-significant correlation *r* = −0.13, *t*(16) = −0.53, *p* = 0.60. The presented results seem to be roughly in line with previous studies using the method of adjustment.

### Task 3: Triadic task

### Method

#### Apparatus, stimuli, and design

The apparatus and the stimuli were similar to Task 1. The main change was the task and response. In each trial, a fixation point at 0°, 0.9°, 2.2°, 5.4°, or 13.9° was shown on a computer screen, visible only to the looker, who fixated on the fixation point when the observers had closed their eyes. Importantly, however, the observers’ task was not to indicate whether they are looked at or not, but where the looker is looking. To that end, a scale was fixed to the top of the monitor. Furthermore, the rectangular area under the scale was hidden by a cladding. The cladding was intended to cover the entire back of the screen as well as the space beside the screen up to the left wall of the room to prevent that observers could guess were the monitor ends. Without the cover, observers might have concluded that the looker’s digitally displayed fixation points could only cover gaze directions that end within the monitor frame. To avoid ceiling effects due to the observer’s perception of the monitor’s size, the monitor as well as the space between the monitor and the wall were covered. The cladding was present in all tasks, only the meterstick was added additionally to the screen for the present task.

**Fig. 4:**
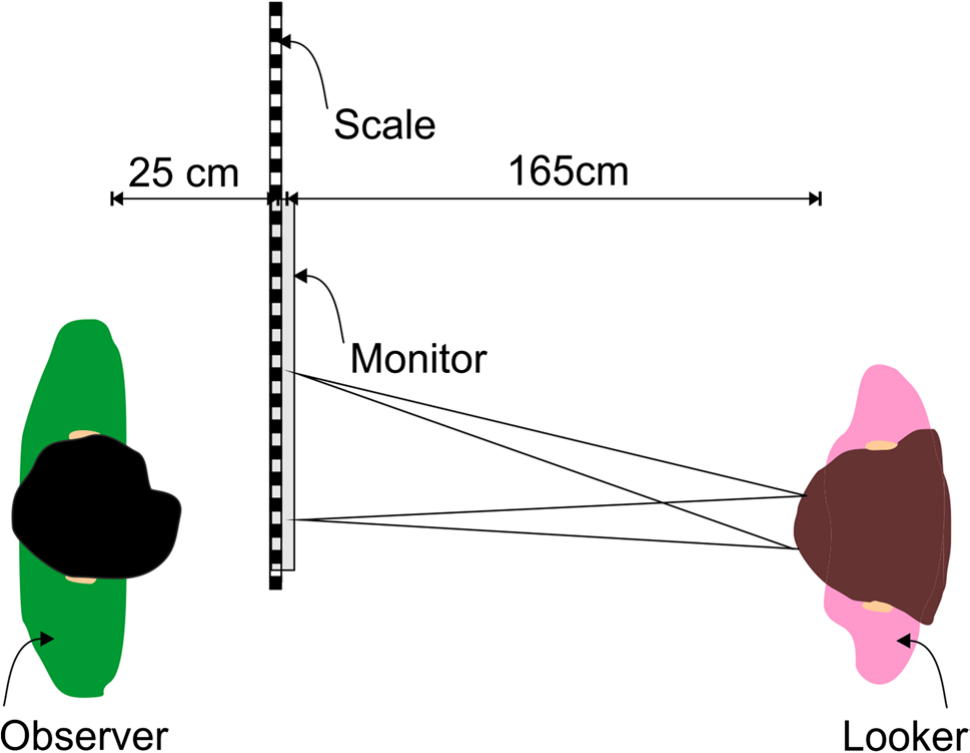
An illustration of the experimental setup for Task 3. Note that the scale was attached to the top of the monitor, and a cladding that reached from the scale down to the table on which the monitor stood hid the extension of the monitor from the sight of the observer

In this task, observers sat at a distance of 25 cm from the screen in order to be able to see the scale (see Fig. 4). The screen was 165 cm away from the observer; thus, the overall distance between observer and looker was 190 cm. The observers were asked to judge which position the looker is fixating on by reading the corresponding (full) cm value on the scale. Between the trials the observers were asked to close their eyes. After the looker had adjusted her gaze to the next fixation point, the observers were allowed to open their eyes again.

### Results and discussion

#### Linear fit

**Fig. 5:**
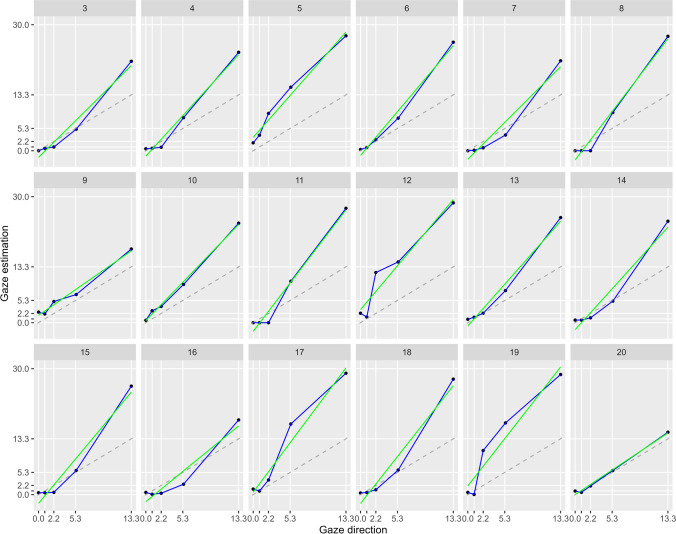
Linear and piece-wise regression for observers. Dots indicate data points, while the coloured lines indicate the piece-wise regression function (blue, sectioned line) and the linear regression (green, straight line). Dashed lines indicate a perfect estimation. Data points under the dashed line indicate underestimation, dots above the dashed line indicate overestimation. (Colour figure online)

As a first approach, we fitted the data to a linear function as researchers have done before (Anstis et al., 1969; West, 2010, 2011). The average slope was 1.8, which was significantly different from zero, *t*(17) = 23.05. *p* < .001, and the average intercept was −0.37, which was not different from zero, *t*(17) < 1. Figure 5 gives an overview of the linear fits.

#### Nonlinear fit

An inspection of the data revealed that they may not perfectly correspond to a linear fit. Actually, almost all of the individual data appear rather nonlinear (see Fig. 5) and change their slopes with exceeding eccentricity of the looker’s fixation point. However, observers showed different patterns of slope changes, which did not easily suggest a commonly used nonlinear function (i.e., cumulative Gaussian, exponential, etc.). Therefore, we decided to calculate a piece-wise linear regression. We defined the changing points to be our measured gaze directions, which resulted in a four-piece linear regression. Thus, the first section of linear regression was between 0° and 0.9°, the second section between 0.9° and 2.2°, the third section between 2.2° and 5.4°, and the fourth section between 5.4° and 13.9°. The four-piece linear regression was conducted individually for each observer. Therefore, for each observer and each section (first to fourth) a linear regression was conducted. This results in four linear regressions for every observer reflecting the four-section structure. We extract the intercept and slope for each section individually for each observer.

The mean intercept of the first section was 0.76, while mean slope for the first section was 0.18 yielding an underestimation on average. The second section (intercept = −0.58, slope = 1.71), third section (intercept = −0.57, slope = 1.70), and fourth section (intercept = −2.01, slope = 1.97) indicated an overestimation on average.

To test whether the slopes are different from each other, we conducted a one-way repeated-measures analysis of variance (ANOVA), with the slopes as the dependent variable and the section as the independent variable. The ANOVA revealed a significant difference between the slopes, *F*(4, 51) = 5.24, *p* = .003, ꞃ^2^ = 0.20, indicating that the slopes change and that the relation between gaze and judgement is not linear. Post hoc *t* test revealed a significant difference only between the slope of the first section and all other slopes, first slope vs. second slope: *t*(17) = −2.23, *p* = 0.04, *d* = −0.83; first slope vs. third slope *t*(17) = −5.18, *p* < .001, *d* = 1.68; first slope vs. fourth slope *t*(17) = −7.89, *p* < .001, *d* = −2.51).

## Comparison between measures

The method of adjustment measured a 1.6 times larger threshold of direct gaze than the method of constant stimuli (5.2° > 3.3°), *t*(17) = −4.83, *p* < .001, *d* = −1.55. The correlation between thresholds was small, *r* = 0.03, *t*(16) < 1. Correcting the measured thresholds with the classical linear overestimation regression revealed estimates of 8.99° and 5.64°, for the method of adjustment and the method of constant stimuli, respectively. We were also interested in how the overestimation effects found with the piece-wise linear regression impacts on the thresholds. The problem of course is that a nonlinear function does not provide a single gain factor; rather the slope of the function changes with the sections of the piece-wise linear regression. We therefore used the individual fittings to obtain the regressions slope at the specific individual thresholds. To do so, we calculated the corrected threshold by using the corresponding linear function of the section. For example, for a participant with a threshold of 3° we would use the segment between 2.2° and 5.4°. To calculate the corrected thresholds, we inserted the threshold of direct gaze in the regression equation (corrected threshold = slope × threshold of direct gaze + intercept). The resulting individualized estimates were used to report corrected thresholds. Please note that with a piece-wise linear regression not only the slope changed within participants, but also the intercept. The intercept ranged on average between −0.57 in the first section to 2 in the fourth section. Therefore, we calculated the corrected threshold not only by using the slope of the corresponding regression, but also the intercept.

**Fig. 6:**
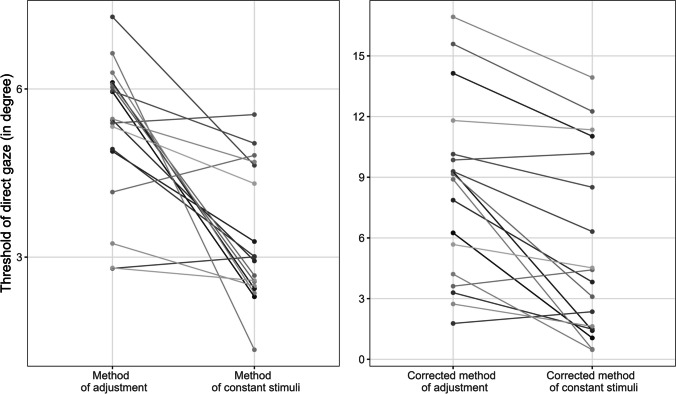
Individual thresholds of direct gaze (dots) and the relation of order (lines) between the method of adjustment and the method of constant stimuli before (left) and after (right) correction with estimation bias of the triadic measurement task

The average estimation bias for the thresholds obtained by the method of adjustments was 1.85, while the overall estimation bias for the method of constant stimuli was 1.57. While numerically different, the estimation bias did not differ significantly from each other, *t*(17) = 1.19, *p* = 0.25. Recalculation of cone-width values revealed an increase of the threshold of direct gaze. The threshold measured with the method of adjustment increased significantly from 5.24° to 8.36°, *t*(17) = 3.14, *p* = 0.006, *d* = 0.86. The threshold measured with the method of constant stimuli increased significantly as well from 3.33° to 5.46°, *t*(17) = 2.22, *p* = 0.041, *d* = 0.51. After the corrections, the thresholds in the two tasks remained to be significantly different from each other, *t*(17) = 4.56, *p* < .001, *d* = 0.64. The corrected threshold from the method of adjustment was 1.5 times larger than from the method of constant stimuli, which was only slightly less than the original difference between methods (1.6 times larger before correction). Corrected thresholds of the method of constant stimuli and the method of adjustment had a high correlation, *r* = .81, *t*(16) = 5.71, *p* < .001 (see Fig. 6).

## Discussion

When measured with the method of adjustment, the threshold of direct gaze is larger than when measured with the method of constant stimuli. Overall, the measured thresholds are in good agreement with the literature, being around 5°. We also found a substantial overestimation of 80% on average using Anstis et al.’s (1969) matching task for gaze direction; it might be argued, however, that the classical linear fit is not completely adequate because it does not consider that for most participants there was little overestimation near perfectly straight gaze, and that the shape was more sigmoid than linear for many observers. We used piece-wise linear regression to accurately estimate overestimation factors for the different sections (see also the General Discussion, for further discussion).

Assuming that the overestimation factors and the thresholds can be reasonably related implies that the zone in which observers perceive to be looked at was 1.6 to 1.8 times as large when the perception of space is concerned than when the objective rotation of the eye is concerned. According to the gaze cone concept, the area of direct gaze can be represented as a wedge or isosceles triangle originating at the looker (Gamer & Hecht, 2007). This concept implies that at all distances between the looker and the observer there is a considerable range of positions around the looker–observer axis where the observer perceive direct gaze. It is this range of positions that may be related to the overestimation factor, and implies that this range is 1.6 to 1.8 times as large as implied by the gaze angle.

The correlation of cone width between the methods of constant stimuli and of adjustment was rather small, which indicates that the order of the participants in each of the two tasks was essentially random. By correcting with the estimation bias the correlation between measurement methods reappears although in average the majority of observers overestimated gaze direction in the area of the threshold of direct gaze. However, the original correlation of noncorrected values is mainly influenced by the reliability of our measures, which might not be optimal here. In order to set up an experiment with a live looker and three tasks, we had to find a compromise between the number of repetitions in the tasks and the total duration of an experimental session. The compromise was primarily designed to obtain data on the parameters of cone size but might have been insufficient for correlations between the measures. Furthermore, our real looker may vary unsystematically in her looking behaviour causing more variance than a computer avatar or photographed stimuli. Recall that the upper limit for a correlation is the square root of the product of the reliabilities (Crocker & Algina, 1986; Onwuegbuzie et al., 2005).

Therefore, with Experiment 2, we would like to replicate the difference in average cone size as well as to review the correlation results. By increasing the trial number as well as the number of participants and additionally conduct the experiment with a much more controllable stimulus — a computer avatar — we improve reliability and power to be able to test about the correlation between the method of constant stimuli and method of adjustment.

## Experiment 2

Experiment 1 revealed a difference between the threshold sizes obtained by the method of constant stimuli and of adjustment. Experiment 2 replicated Experiment 1, with a number of changes that were intended to reduce noise and improve measurement precision. First, a computer avatar replaced the live looker, thus eliminating noise in the stimulus. Second, the number of trials was increased to reduce noise in the dependent variable. Third, the threshold was tested on both sides.

### Participants

Twenty observers (3 male, 17 female), aged between 20 and 43 years, volunteered for course credit or candy. Sample size was chosen due to a large effect size in Experiment 1. All observers had normal or corrected-to-normal visual acuity and intact color vision. They gave written informed consent before participation. The experiment was approved by Bielefeld University’s ethic committee, and it conformed with the Helsinki protocol.

### Task 1: Method of constant stimuli

### Method

#### Apparatus and stimuli

A computer-generated avatar was presented on a 36.4 cm × 27.7 cm-sized Sony Multiscan G420 monitor with a frame rate of 89 Hz. The display had a resolution of 1,280 × 1,024 pixels. The stimuli were presented in full screen mode. The width of the avatar’s head was 16.5 cm and the height 25.8 cm. The interpupillary distance was 6.5 cm. The screen size of the virtual head approximately equaled that of an adult human head. The virtual head was generated based on a modified Sims (Die SimsTM 4, Electronic Arts GmbH) avatar, in which both eyes were cut out and replaced by transparent pixels. The simulated eyes were created and controlled by a custom written Python script as simulated spheres. The relative sizes of the eyeball, iris, and pupil were based on normative data so that the iris was 30% of the eyeball size, and the pupil was 10% of the eyeball size (Gharaee et al., 2014; Sanchis-Gimeno et al., 2012). The simulated eyes were controlled independently in their horizontal rotation. Accordingly, it was possible to adjust the vergence angle between the eyes in addition to the nominal rotation angle. The avatar mask was laid over the simulated eyes. Pictures were generated that showed the avatar’s face with a range of gaze directions by rotating the simulated eyes in the horizontal plane. A rotation of 15° to the left as well as to the right was used as the widest eye rotation. Gaze direction was varied in steps of 3° from 15° rotated to the left up to 0°, as well as from 15° rotated to the right up to 0°, resulting in 11 gaze directions. Here and in the following, gaze direction will be described from the observer’s perspective, and gaze to the left will be labelled as negative values. For this experiment, the vergence eye angle was fixed to the natural vergence at the presentation distance, which was 1.1° per eye for a distance of 165 cm. No angle kappa was applied (see Linke & Horstmann, 2024 for further information). Presentation distance was chosen to match Experiment 1.

#### Design and procedure

Eleven gaze directions between −15° and 15° in steps of 3° were presented to the observer. Observers watched the stimuli as long as they wanted and then judged whether the computer avatar was looking at them by pressing the left arrow key for “yes” or the below arrow key for “no”. There was no time limit for the answer. After pressing the key, a fixation cross appeared in the middle of the screen for one second, followed by the next gaze stimulus, which was presented without a time limit. Each of the 11 gaze-direction conditions was repeated 35 times, resulting in 385 trials. The order of the trials was random, with a new random sequence generated for each observer. The observer performed two practice trials at the beginning.

### Results and discussion

**Fig. 7:**
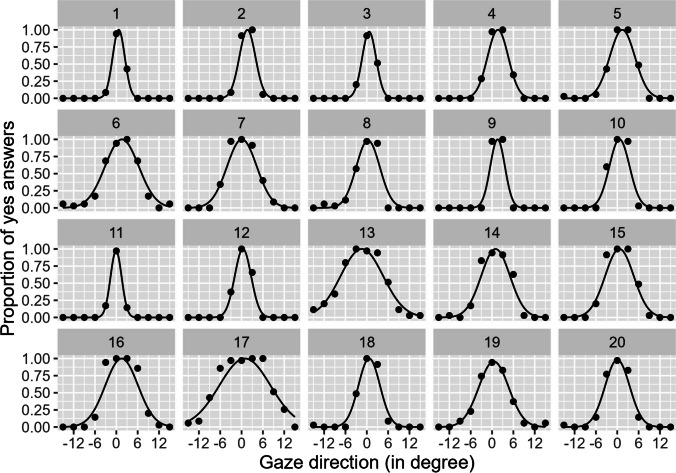
The individual proportions (points) for judgements of being looked at and the fitted Gaussians (lines). The *x*-axis depicts gaze direction; the *y*-axes the proportion of direct gaze judgments

A Gaussian was fitted to proportion yes for each participant. The threshold of direct gaze was indicated by the standard deviations of the individually fitted gaussians. On average the threshold of direct gaze was 3.51° (see Fig. 7) with an inter-observer standard deviation of 1.44°. The center of the Gaussian was slightly shifted to the right, which indicates that a gaze of 0.69° to the right is judged as perfectly being looked at.

### Task 2: The method of adjustment

### Method

#### Apparatus and stimuli

Apparatus and stimuli were similar to the corresponding condition in Experiment 1. Instead of fixed gaze directions, the observers were now able to change the gaze direction of the computer avatar by scrolling their mouse wheel. The range of gaze directions was the same as in the method of constant stimuli (−15° to 15°) but in steps of 1° observers were able to change the gaze direction of the computer avatar.

#### Design and procedure

As in the first experiment, there were ascending and descending series. Ascending series started with an extreme horizontal rotation (15° either to the left or to the right) and could be adjusted in direction of 0° gaze direction. Descending series started with 0° gaze direction and could be adjusted up to 15° horizontal rotation. In contrast to Experiment 1, half of the trials were concerned with the left side of gaze, whereas the other half was concerned with the right side of gaze. Note that when the instructions for a trial specified the right (left) side of gaze, gaze could be adjusted between 0° and 15° on the right (left) side only. The combination of the factors side and series resulted in two types of trials for the left side and for the right side. The first condition for the left side combined the instruction “just looked at” with using ascending series that started with eyes rotated to 15° to the right. The participants then adjusted horizontal eye rotation to the left side in steps of 1° until that point where they perceived gaze as just directed at them. The maximum possible value was 0° (straight gaze). The second condition for right side series combined the instruction “no longer looked at” with descending series with eyes starting at 0° straight gaze; horizontal eye rotation could be adjusted to a maximum of 15° gaze directions to the right. The same procedure was used for the left side (here and in the following, we denotate left sided rotations with negative numbers). The first condition of left side series combined the instruction “just looked at” with ascending series, starting with eyes rotated −15° and could be adjusted in steps of 1° until the 0° gaze direction. The second condition for the left-side series combined the instruction “no longer looked at” with descending series, starting with 0° gaze direction and could be adjusted to −15° orientated eyes to the left.

Presentation and response registration were controlled by a custom written Python script using routines from PsychoPy (Peirce, 2008). The instruction for a given trial preceded each trial and was visible in a shortened version while performing the task. The adjustment of the eyes was made by using the scroll wheel of the mouse. By scrolling upward, the gaze was shifted to the right, while scrolling downward led to a shift to the left. The observers could adjust the eyes as long as they wanted to; they confirmed their final result by pressing the “enter” key on the keyboard. Two warm-up trials were made before the experiment proper, during which the researcher probed the participants’ understanding of the task and provided answers to possible questions. Each combination of conditions was repeated 15 times resulting in a 2 (side) × 2 (series) design with a total of 60 trials. The order of the trials was random, with a new random sequence generated for each observer.

### Results and discussion

**Fig. 8:**
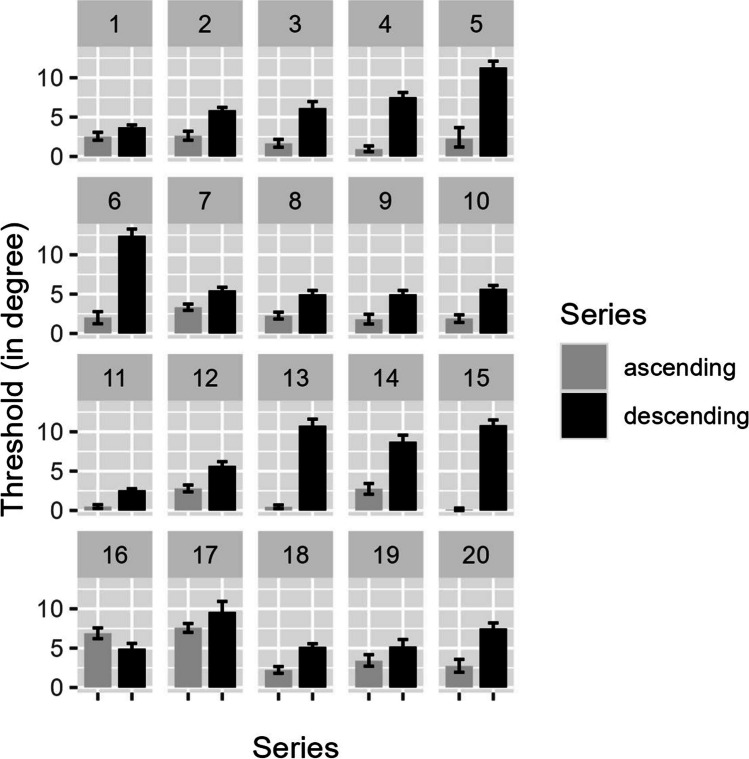
Individual thresholds for ascending and descending series with standard errors per observer and series

The data were aggregated for each observer and each task and side separately, resulting in four means per observer. Thresholds were determined by averaging of ascending and descending series. Left-side series yielded a mean of -1.97° for ascending series and a mean of −6.37° for descending series. The overall mean for the left side was −4.35°. Right-side series yielded a mean of 2.89° for ascending series, and 7.31° for descending series. The overall mean of the right side was 5.31°. Within-subject standard deviation was highly variable ranging from 0.82° to 7.57°, with an average of 3.25° (see Fig. 8). The overall mean averaged over both series, and sides resulted in a threshold of direct gaze of 4.73°. *T* tests indicated a significant difference between series, *t*(17)= −5.74 , *p* < 0.001, *d* = 1.88, indicating some hysteresis. Ascending and descending series were weakly and nonsignificantly correlated, *r* = −0.10, *t*(19) = −0.46, *p* = 0.65. The presented results of the threshold of direct gaze seem to agree with other results using the method of adjustment.

## Comparison between tasks

**Fig. 9:**
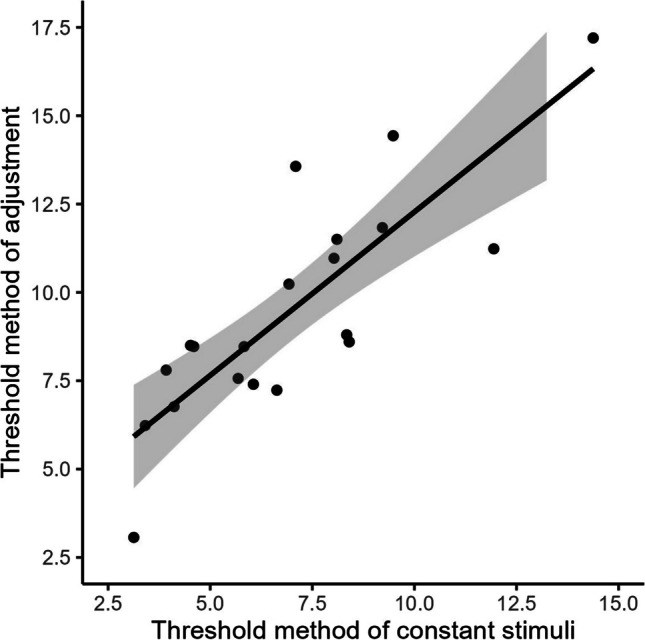
Correlation between method of constant stimuli and method of adjustment of Experiment 2. Dots indicate thresholds of the measures in degree; the line indicates correlation between measures

Thresholds measured with the method of adjustment were again larger than when measured with the method of constant stimuli (4.7° > 3.5°), *t*(19) = −6.32, *p* < .001, *d* = −0.81. The correlation of the individual thresholds between the two tasks was high, *r* = 0.83, *t*(18) = 6.34, *p <* .001(see Fig. 9).

## General discussion

The aim of this study was to compare and relate the results from different experimental tasks that measure gaze. In particular, we compared the results from the method of adjustment (an adaptation of the method used by Gamer & Hecht, 2007, and others) and from the method of constant stimuli (an adaptation of the method used by Gibson & Pick, 1963, and others), which both are assumed to measure the cone of gaze. In addition, we were interested in relating the results from a triadic task (first introduced by Anstis et al., 1969) that measures the perception of gaze direction independently from the question of direct gaze (the perception of being looked at) with the two measures of gaze cone.

With regard to the first aim, we find that the method of adjustment yielded larger thresholds of direct gaze than the method of constant stimuli. This supports the impression derived from the literature. Importantly, however, comparisons between studies in the literature are risky because these studies vary on variables that plausibly influence the measure, in particular the looker identities and the used distances. Moreover, Lobmaier et al. (2021) found that there are considerable differences between observers in measures of gaze cone, and, given that the observer samples are often not particularly large, it is possible that comparisons between studies fall prey of sample effects. The present study eliminates these ambiguities by measuring the gaze cone with the two approaches in the same setting with the same looker, and same observers.

As the task introduced by Gamer and Hecht (2007) uses the method of adjustment and the task introduced by Gibson and Pick (1963), the method of constant stimuli, one is tempted to attribute the differences between these two types of methods. It is common wisdom that when in doubt, the method of constant stimuli is to be preferred, as this method is considered least susceptible to effects of bias and expectation. We have used different starting points (i.e., ascending and descending series) for the method of adjustment to eliminate symmetric biases (such as hysteresis as caused by conservative vs. lenient response criteria) and thus consider the point of symmetric bias not relevant here. However, it is certainly possible that asymmetrical biases play a role, for example, that the perception of being looked at is more persistent and more difficult to disregard than the perception of not being looked at, and that the perception of being looked at does not change easily. This is consistent with the difference and small correlation between our ascending and descending series: Adjusting the eyes to the point where the model is no longer looking at the observer revealed higher thresholds than adjusting to the point where the model starts to look at the observer. An asymmetrical bias may thus be a plausible explanation for the gaze cone being 20–40% larger with the method of adjustment than with the method of constant stimuli.

Moreover, one might argue that the method of constant stimuli uses direct gaze as the reference category (Say “yes” when you are being looked at), which is only the case in the ascending task (Stop when you are just being looked at), but not in the descending task, which uses not being looked at as the reference (Stop when you are no longer looked at). This would imply that only the ascending series from the method of adjustment are comparable with the method of constant stimuli. It would be another argument for the point that the results depend on the exact task and context.

What does this difference imply? If two measurement methods are parallel or alternate forms of each other, they should have similar psychometric properties (e.g., means, standard deviations; Murphy & Davidshofer, 1988). The difference in averaged gaze cone size implies that these two tasks are not exactly parallel but partly measure different things. One might note that previous examinations found similar results with other types of judgment (Chen et al., 2019; Morgan et al., 1974), indicating that this is not a particular problem of gaze perception.

A second set of results concerns the correlations between the measures. In Experiment 1, not only did the means from the two tasks not match, but they also did not correlate strongly with each other. In contrast, Experiment 2 found a large correlation between the two tasks. According to Fine (1992) a high correlation between alternative measures of the same construct is a necessary condition for the methods measuring the same construct. Recall that a high correlation of course does not require identical thresholds obtained with the two tasks: All that is required for a high correlation is that the order of the thresholds within the two tasks is the same.

A primary reason for low correlations in Experiment 1 is unreliable measurement. The lack of reliability attenuates a possible correlation as the correlation between two measures cannot exceed the square root of the product of their reliabilities (Crocker & Algina, 1986; Onwuegbuzie et al., 2005). In order to deal with the three measurements in a single session, we kept the tasks short, and possibly this led to more variability in the measurement than expected. Noise in the stimulus may also contribute to variability in the measurement, as our live looker may have had some uncontrolled variation in his gazing behaviour. For example, a real person may move or change their expression or slightly turn their face up or down providing additional visual cues that are not present in a static computer-generated face that is always perfectly still. This additional noise may have reduced reliability.

Second, this might affect the method of adjustment more than the method of constant stimuli when using a live looker in contrast to a computer avatar. In the method of adjustment, the live looker has to follow the dot on the screen. The position of dot is freely adjusted by the observer, and therefore not only the position of the dot changes unpredictably but also the velocity of the position change is determined by the observer (how fast the observer scrolls with the mouse wheel). In contrast, in the method of constant stimuli, the dot is static. It is reasonable to assume that the difference in the difficulty of the task affects the looker’s accuracy of fixating and introduces much more uncertainty and variability in the method of adjustment, which causes additional noise in the live looker condition. When using a computer avatar, we have a much more controlled stimulus, and therefore reduced noise in the answers.

A third point of discussion is the triadic measurement of gaze perception accuracy in Experiment 1. Consistent with the literature, we found that gaze direction is overestimated. In contrast to previous studies (Ando, 2002; Anstis et al., 1969; West, 2010, 2011), the relation between actual and perceived gaze showed a nonlinear pattern of estimation accuracy. As in some previous studies that did not use linear fittings (see Gonzalez-Franco & Chou, 2014; Masame, 1990), we also found not only an overestimation bias but also an underestimation bias, depending on the fitted range of gaze directions.

On average, the estimation bias of the triadic measurement method was smallest near zero. It may be that the first gaze direction (0.9°), and maybe the second gaze direction as well (2.2°), were difficult to discriminate from the zero-degree gaze direction for the observers. The overall accuracy threshold of gaze direction deviation seems to be between 1.3° and 2.8° described as the amount of shift that this represents at the target (see Gibson & Pick, 1963; Symons et al., 2004). Therefore, a shift of the target of only 0.9° or even 2.2° could be too small to be perceived. In contrast, Symons and colleagues (2004) have shown a remarkable precise perception of target shift for a target located between the observer and the looker. In their study, visual acuity for gaze directions of 0° and 0.87° was between 0.3° and 0.5°, indicating that a gaze direction of 0.87° should be distinguishable from 0° gaze direction. Nevertheless, naturally occurring differences in visual acuity between observers may be a reason for an underestimation of the first and partly second regression section and could therefore have caused the sigmoid shape. This indicates that the linear fitting may be inaccurate for values nearby 0° but may be precise and fitting for gaze directions higher than the individual visual acuity.

Another possible explanation is the so-called social-interaction bias firstly introduced by Masame (1990). He claims that gaze directions near the observer’ face tend to be perceived as being egocentric and, therefore, perceived as being directed to the face of the observer's. Following Masame, the perception of being looked at influences the judgements of the gaze direction in the near face area. Although the observer’s task is to estimate the crossing of the gaze vector with the meterstick in front of him, Masame postulates that nevertheless, the position of the observer right behind the scale influences the triadic gaze perception. Both approaches may explain the significant difference between the first section slope and the other sections of the piece-wise linear regression, while the slopes of the other sections (second to fourth) are similar.

Furthermore, we asked whether the perception of gaze direction from the triadic task is linked to the perception of direct gaze from the dyadic tasks within an observer. Unfortunately, the nonlinear properties of the overestimation factor make a meaningful interpretation of the correlation between threshold size and overestimation bias nearly impossible. The variable overestimation gains in the nonlinear data result in larger slopes for more extreme rotated gaze directions. In case of our four-piece linear regression, we have small slopes for the first section indicating even an underestimation and a gain of overestimation for the second, third, and fourth section with the fourth section reaching the largest overestimation. Now we would like to know whether the individual threshold of direct gaze is linked to the individual overestimation. This could be analyzed by choosing the slope of the section of the four-piece linear regression, in which the threshold of direct gaze is localized, and correlate it with the threshold of direct gaze. For example, if an observer had a threshold of direct gaze of 3°, we would use the individual slope of his third section regression because it models the data between 2.2° and 5.4°; another observer could have a threshold of 6°, and we would use the individual slope of their fourth section regression. Therefore, the threshold of direct gaze directly influences the chosen section of the regression. When keeping in mind that the slopes vary systematically, with the different sections of the regression with slopes being larger for the later sections (as they reflect more extreme gaze directions), there is a clear confounding between regression section selection and possible systematic correlation between threshold sizes and overestimation sizes. Positive correlations between the threshold of direct gaze and the classical overestimation bias could therefore just arise due to the variable overestimation gain within observers rather than reflecting a link between direct gaze and gaze direction perception.

Nevertheless, we were able to correct the thresholds of Experiment 1 by determining the over- or underestimation by using the full regression equation of the section that corresponds to a given threshold for a given person. For most participants, there were overestimations of gaze direction at the thresholds of direct gaze, and thus the corrected threshold sizes were higher than the uncorrected ones. Nevertheless, in some cases, where the threshold of direct gaze was quite small, the threshold got smaller due to the correction. It is somewhat counterintuitive that a small cone should get even smaller with correction, while a large cone gets wider with correction. Therefore, it might be questioned whether the results of the triadic measurement method (a measurement of gaze direction) can be applied to a specific value as the threshold of a dyadic measure (a measurement of direct gaze).

How gaze direction and direct gaze are perceived is a current topic of debate. Some approaches suggest that gaze direction is perceptually derived from the luminance distribution of the eyes. According to this explanation, a direct gaze here would be perceived when the pupil-iris complex and the sclera on the left and right side are arranged symmetrically (Ando & Osaka, 1998). Whenever arrangement is not symmetrical, the gaze will be perceived as averted. Therefore, it is possible that a perception of gaze direction is not necessary for the perception of direct gaze, and that direct gaze is perceived directly based on the stimulus configuration (and not on perceived gaze direction). On the other hand, the geometrical approach (Palmer et al., 2020; Todorović, 2006) would suggest a quite different sequence of events. The geometrical approach proposes that gaze direction is determined by the perceived rotation of the eyeball. Because the real rotation of the eyeball is not available for the observer, the pupil eccentricity in the visible eyeball is used as a cue for the real rotation. If the rotation estimation (so, in fact, the perceived gaze direction) agrees with the observers criterium of direct gaze, the gaze will be judged as being direct. The luminance distribution account and the geometric account imply different answers to the question whether a triadic estimation bias can be applied to a dyadic task. In conclusion, we cannot give a certain answer about the relation between gaze direction and direct gaze.

In summary, both experiments indicate a stable difference in mean thresholds of direct gaze when measured with the method of constant stimuli and the method of adjustment. This difference has been demonstrated with a real-live looker as well as with a computer avatar. Thresholds are somewhat smaller with the method of constant stimuli than with the method of adjustment. This difference may be due to the prominence of the direct gaze category relative to the averted gaze category.

## Appendix 1

**Table 1:** Overview of the size of the area of direct gaze by measuring method

Author	Method	Distance in cm	Size of the area of direct gaze in degree
Gibson and Pick, 1963	Method of constant stimuli	200	6
Lobmaier et al., 2021	Method of constant stimuli	60	5
Balsdon and Clifford, 2018	Method of constant stimuli	57	9
Gamer and Hecht, 2007	Method of adjustment	100, 500	8-9
Gamer et al., 2011	Method of adjustment	100	11
Harbort et al., 2013	Method of adjustment	100	14
Horstmann and Linke, 2021	Method of adjustment (modified)	160, 220, 300, 420, 575, 790	5
